# Native Label‐Free Spectral Biochemical Fingerprinting of an Enveloped Mammalian RNA Virus Reveals Mode of Action of a Virucidal Drug

**DOI:** 10.1002/smtd.202502015

**Published:** 2026-08-11

**Authors:** Denis Rajnovic, Fatima Matroodi, Lamyaa M. Almehmadi, Tea Carletti, Alessandro Gessini, Igor K. Lednev, Claudio Masciovecchio, Alessandro Marcello, Barbara Rossi

**Affiliations:** ^1^ AREA Science Park Elettra – Sincrotrone Trieste S.C.p.A Trieste Italy; ^2^ International Centre for Genetic Engineering and Biotechnology (ICGEB) Trieste Italy; ^3^ AREA Science Park Trieste Italy; ^4^ Department of Materials Science and Engineering Massachusetts Institute of Technology Cambridge Massachusetts USA; ^5^ Department of Chemistry University at Albany, State University of New York SUNY Albany New York USA

**Keywords:** antiviral mechanism of action, bottom‐up and top‐down DUVRR virus workflows, deep UV resonance Raman spectroscopy (DUVRR), label‐free biochemical fingerprinting, spectral analyses, stoichiometric reconstruction, vesicular stomatitis virus (VSV)

## Abstract

Pandemic preparedness demands the availability of state‐of‐the‐art tools for fundamental research on viruses to support rapid development of antiviral and prophylactic interventions. Virion composition is currently studied by a limited number of disruptive techniques, while here, a novel workflow for non‐invasive and label‐free biochemical fingerprinting is presented. Deep‐Ultraviolet Resonance Raman spectroscopy is employed as the primary analytical tool, while stoichiometric‐based spectral reconstruction and multilinear regression analysis are used to perform bottom‐up and top‐down biochemical characterization of the vesicular stomatitis virus (VSV) in physiological‐like conditions, respectively. Raman spectra from virions and single biochemical components (lipids, amino acids and nucleotides) excited at 213 nm are used to disentangle the virus biochemical composition and to probe the mechanism of action of a lipid‐disrupting virucidal drug. Available biochemical and sequence information together with single‐component spectra allows the reconstruction of the full virion spectrum with excellent accuracy. Altogether, this strategy of virion biochemical fingerprinting paves the way to a better understanding of virus composition and mechanisms for inactivation.

## Graphical abstract

Deep‐Ultraviolet Resonance Raman (DUVRR) spectroscopy is introduced as a label‐free, non‐invasive analytical workflow for the biochemical fingerprinting of enveloped viruses. By integrating bottom‐up stoichiometric reconstruction with top‐down biochemical extraction, this strategy accurately disentangles complex viral compositions in physiological‐like conditions. This approach reveals the mechanisms of virucidal drug action, offering a powerful tool for rapid pandemic response and fundamental virology research.

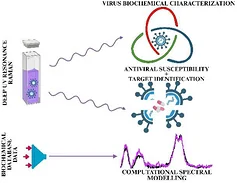

## 1 Introduction

Innovations in biochemical analysis of viruses and antiviral susceptibility studies have become more and more relevant in the context of the increasing frequency of emerging pathogens with pandemic potential [1, 2, 3, 4, 5]. To improve overall pandemic preparedness and countermeasures, a simple, fast, inexpensive, and accurate tool that could be potentially implemented in high‐containment biosafety level 3–4 laboratories is needed. Ideally, this tool must be non‐destructive or minimally invasive and label‐free, allowing for subsequent analysis of intact virus samples.

The basic biochemical elements of a virus include the nucleic acid genome, the protein capsid, and, for enveloped viruses, the lipid peri‐capsid. Currently, available methods used to characterize the building blocks of a virion are next‐generation sequencing [6], mass spectrometry [7], cryo‐EM [8], X‐ray crystallography [9], NMR spectroscopy [10], electrophoresis [11], chromatography [11], and serological assays [11]. Despite being well‐established, many of them are labor‐intensive, destructive, require labelling, specialized training, and laboratory environments, and are resource‐intensive; thus, these techniques are ill‐suited to provide a rapid response. Raman spectroscopy, a light scattering technique, offers solutions to these challenges by providing molecular‐level information about the biochemical components of a specimen through the analysis of their vibrational spectra [12, 13]. The high effectiveness of this technique for delivering a biochemical fingerprint of complex specimens, even in physiological‐like conditions, in a non‐invasive way and without the need for chemical labeling is particularly appreciated for viral diagnostics [14, 15]. It has been recently proved that an accurate virus identification can be provided by Raman signatures in real time and label‐free mode [16]. The potential of Raman spectroscopy can be further explored to enhance the sensitivity and specificity of virus detection thanks to the resonance Raman effect [17]. In the Deep UV Resonance Raman (DUVRR) spectroscopy, the excitation wavelength is chosen to coincide (resonance) with an electronic absorption band of the molecules of interest. In this condition, the resonance produces a great enhancement (up to 10^6^) of the Raman signal associated with the part of the molecule on which the electronic transition is centered [17, 18]. While the vibrational spectra of complex biological systems typically exhibit ‘many‐to‐one’ overlaps, DUVRR leverages this selective resonance to prioritize dominant biochemical markers, effectively simplifying these relationships and allowing for a more direct correlation between spectral shifts and specific biochemical classes. Moreover, UV excitation allows for a substantial increase in the efficiency of Raman scattering, resulting in a dramatic increase in the Raman signal. This is due to the fact that the Raman scattering efficiency is proportional to the fourth power of the excitation frequency (I ∝ ν^4^) along with the resonance enhancement. In addition, the absence of fluorescence interference background, specifically with energy of excitation below 250 nm, improves the signal‐to‐noise ratio of Raman spectra. DUVRR spectroscopy provides a valuable tool to selectively separate the vibrational signals of chromophores, which are quite often sites of biological function, such as protein, nucleotide, and lipid components of the virion [19, 20, 21, 22, 23, 24]. Moreover, this technique has proved to be a valuable asset in various research fields both in situ and in stand‐off (remote) measurements such as in analysis of biomolecules (nucleotides [25, 26, 27], proteins [28, 29, 30, 31, 32] and lipids [33, 34]), analysis of bacteria [35, 36, 37, 38, 39] and fungal spore [40], characterization and classification of cancer cells [41], investigation of protein fibrilization [42] and folding [43, 44], DNA methylation [45] and quality control of mRNA vaccines [46, 47].

In the context of pharmacology in pandemic response, there is a constant need to develop new therapeutic options and broad‐spectrum antivirals that can be used against emerging pathogens. Both the identification of new compounds and drug repurposing approaches are critical. In drug‐repurposing screens, therapeutics with clinical data on the effective dose, treatment duration, side effects, and toxicity that are identified as safe‐in‐human drugs can rapidly be translated to patients [5, 48, 49, 50, 51, 52]. Thus, the need to rapidly identify targets and mechanisms of action of drugs is of great importance to effectively contain pandemics. Until now, DUVRR spectroscopy have been proven as a valuable tool in various pharmacokinetics and pharmacodynamics studies: mechanism of action of antibiotic and antibiotic susceptibility in bacteria [53, 54], drug repurposing strategy for RNA G‐quadruplex binders [55], drug‐protein binding systems [56], pharmacokinetics of antibiotics in biospecimens [57, 58], investigation of novel antimalaria drug in biomimetic solutions (mimic drug transport inside malaria‐infected erythrocytes through parasitophorous membranes) [59].

The versatility of DUVRR has attracted growing interest in its application to biological studies, and in our case, particularly in virus research, where its full potential is largely unexplored. With this in mind, we carried out a series of DUVRR tests on biomolecular standards and a mammalian enveloped RNA virus model to design a framework to perform bottom‐up and top‐down biochemical characterization workflows in a label‐free, non‐destructive, and physiological‐like conditions. To the best of our knowledge, we report the first DUVRR spectra of a mammalian enveloped virus supported by experimental and computational spectral interpretation. Our study shows the computed spectral contribution from key biochemical components (lipids, amino acids, and nucleotides). We also devised a workflow for using available database sequences to compute virus spectrum, enabling the determination of biochemical composition from a spectrum by using Multilinear Regression (MLR) analysis. The potential of DUVRR in antiviral research is demonstrated by the study of the mechanism of action of a virucidal drug capable of disrupting the lipidic envelope. This study outlines a framework for virus research using DUVRR spectroscopy, which can be applicable to other types of enveloped and non‐enveloped viruses, to provide a fast response upon the emergence of deadly pathogens.

## 2 Results and Discussion

### 2.1 DUVRR Virus Spectra

**FIGURE 1:**
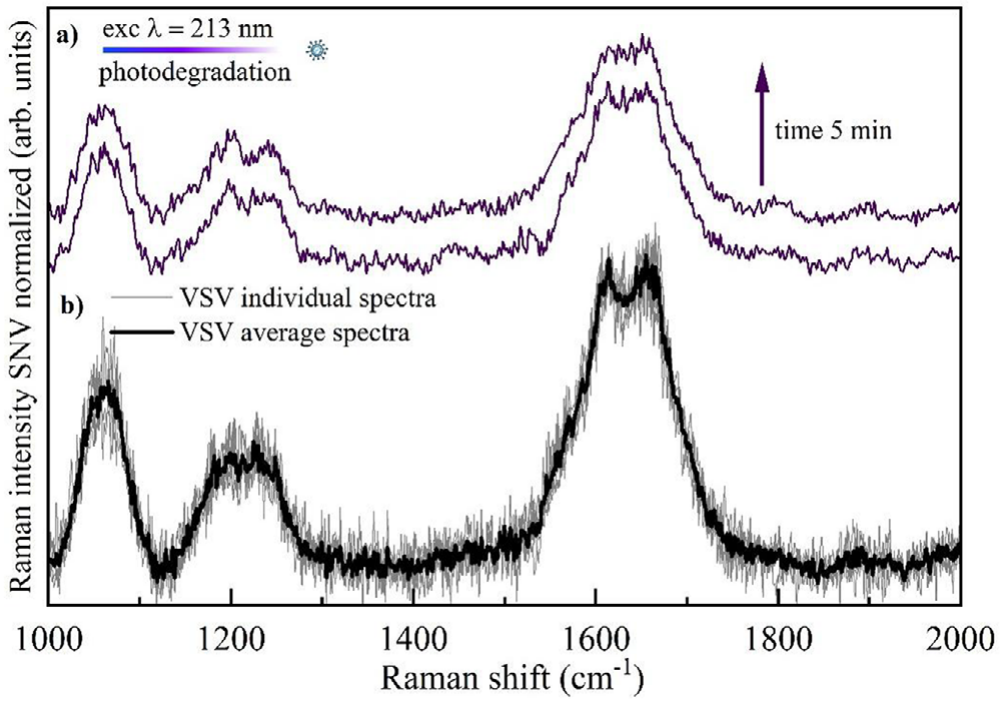
The DUVRR spectra of VSV virus acquired with excitation wavelength at 213 nm. (a) Monitoring of photodegradation during the first 10 min of exposure. VSV spectra collection time was 5 min. (b) The individual VSV spectrum from each batch is present in the graph with grey color, and the spectra average in black color. The number of individual spectra is 5.

The Vesicular Stomatitis Virus (VSV) is an enveloped RNA virus non‐pathogenic for humans that serves as a useful model for viruses causing the most recent viral outbreaks, such as SARS‐CoV‐2, Influenza, and Ebola virus [60, 61]. The DUVRR spectra of VSV in PBS buffer solution at a concentration of 1 mg/mL were acquired with an excitation laser wavelength of 213 nm. After evaluating feasibility with multiple excitation wavelengths, we selected 213 nm as the optimal excitation wavelength due to its significant enhancement of protein and lipidic components, as these biochemical species constitute more than 95% of total VSV mass. Samples were continuously horizontally oscillated during spectral collection to avoid photodegradation. As seen in Figure 1a, virtually no photodegradation of the VSV sample within 10 min of exposure was detected. As a result, DUVRR spectra of VSV virus solution were collected with 5 min acquisition time, which provides faster spectral collection with an S/N ratio of 135. The Signal‐to‐Noise Ratio (SNR) was calculated as the mean peak intensity within the 1580–1680 cm^−1^ region of interest for two consecutively recorded spectra, divided by the standard deviation of these peak intensities. The spectrum of each replicate and the average spectrum of the VSV virus are represented in Figure 1b. In addition, individual spectra of VSV were collected on a different day and from several virus batches with an S/N ratio of 47. To ensure maximum spectral integrity and to reflect the native state of the virion, the individual VSV spectra were collected from *n* = 5 independent biological batches, and only the initial acquisition from each batch was utilized for the final average. Differences between viral replicates were minimal, and an average spectrum of all replicates was used for further analysis. The DUVRR spectral bands of the VSV virus are further discussed in the following section.

### 2.2 Determination of Virus Biochemical Architecture

**FIGURE 2:**
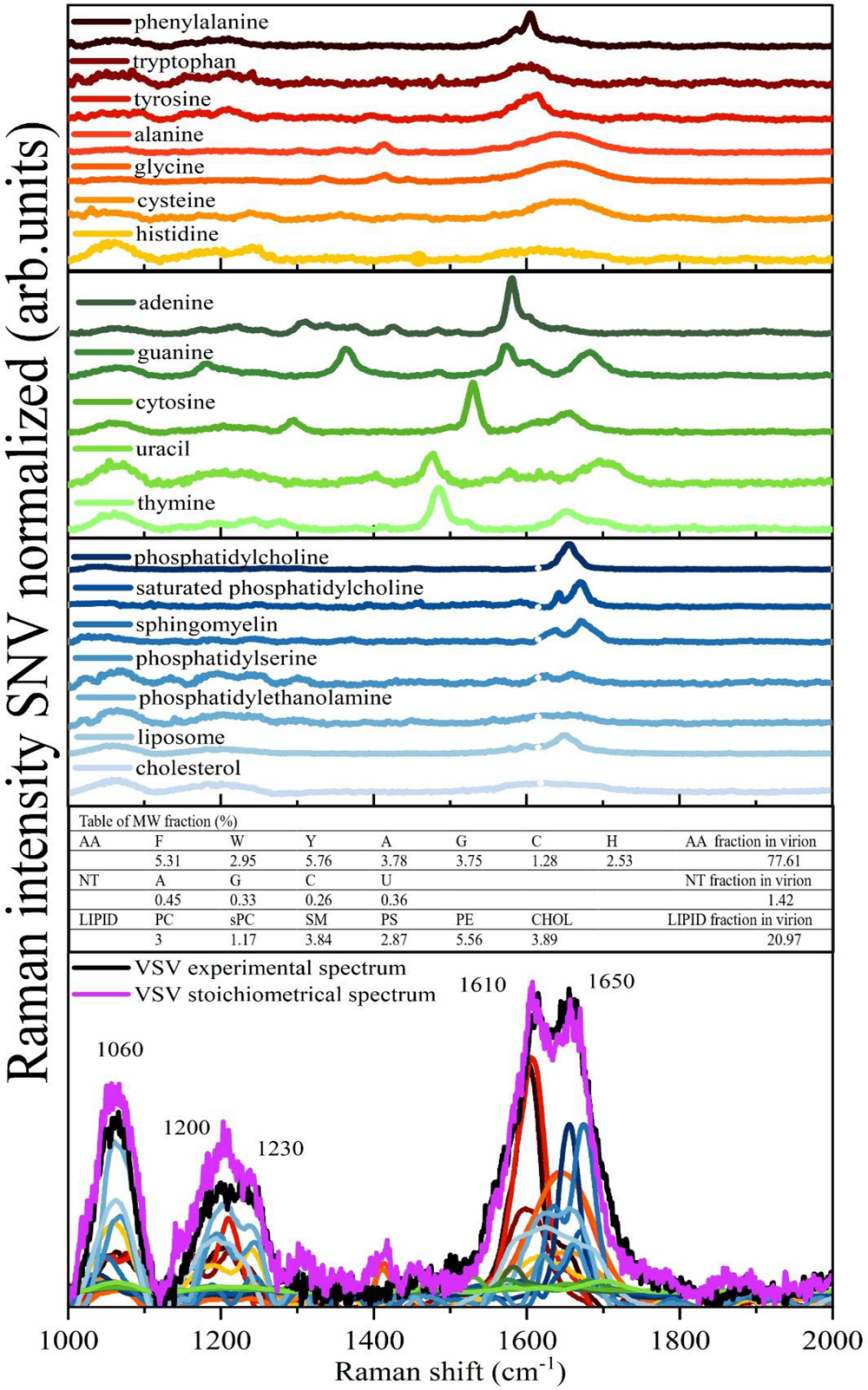
Determination of Virus Biochemical Architecture. The upper panel shows DUVRR spectra of biomolecular standards: amino acids in red, nucleotides in green, and lipids in blue scale. The middle panel represents molecular weight fractions of biomolecules in the VSV virion. The lowest panel shows the DUVRR spectra of biomolecules scaled by their representative molecular weight fraction. The VSV experimental spectrum is presented in black, and the mean of the scaled MW‐biomolecular spectrum is in purple and is defined as the VSV stoichiometric spectrum.

The DUVR spectrum of VSV virus is dominated by peaks at 1600 and 1200 cm^−1^. To identify the spectral contribution of individual biochemical components in the spectral regions at 1600 and 1200 cm^−1^ of the VSV spectrum, we measured individual biochemical components: amino acids, nucleotides, and lipids, that are naturally present in the VSV virion. Each individual component was measured at 10 mM concentration, in separate solutions and under oscillation. In the upper panel of Figure 2, individual biocomponent spectra of amino acids are shown in red scale (a), nucleotides in green (b), and lipids in blue (c). Representative spectra were baseline corrected and standard normal variates (SNV) normalized. The main spectral features of amino acids can be found within the 1550–1750 cm^−1^ range and around 1230 cm^−1^. At 213 nm excitation wavelength, phenylalanine is the most resonant active species among the aromatic amino acids. The peak around 1615 cm^−1^ wavenumber position of aromatic amino acids is attributed to the ring stretching modes [30].

Nucleotides show resonant activity within the full 1200–1800 cm^−1^ wavenumber range, with adenine being the most resonant active one, especially peaking at position 1575 cm^−1^, attributed to CH_2_ scissoring's and vibrational stretching modes of the aromatic ring [62]. This peak can be used as a spectral marker of RNA/DNA presence when analyzing complex biological samples [63]. Lipids are the least characterized element by DUVRR spectroscopy. Lipids used in this work were analyzed individually and in the form of a liposome, which serves as a more realistic model of the viral envelope. Phosphatidylcholine (PC) is the most resonant active species, and the main spectra feature at 1650 cm^−1^ is related to the C═C stretching vibration and indicates the presence of unsaturated fatty acid residues in the PC molecule [64]. On the other hand, when analyzing saturated PC's like DPPC and DSPC (virtually identical spectra), they have two main peaks, one centered at 1640 cm^−1^ and the other at 1670 cm^−1^ wavenumber position. The spectral feature at 1640 cm^−1^ is a marker for saturated fatty acid compounds (C═O bond being involved in the H‐bond). In the presence of unsaturated fatty acid compounds, this peak is overlapped by a much more intense 1650 cm^−1^ band related to C═C stretching vibrations. The peak at position 1670 cm^−1^ can be related to carbonyl stretching of the phosphorylcholine group, as carbonyl stretching of acetylcholine and related compounds have been found in 1660–1680 cm^−1^ wavenumber range [65]. Sphingomyelin (SM), a phospholipid with a similar structure to saturated PC (phosphorylcholine head group and saturated fatty acid residues), has peak positions as those observed in saturated PC's [66]. However, the peak at position 1670 cm^−1^ is broader for SM than in saturated PC. This can be explained by the presence of ceramide backbone in SM, a fatty acid linked by an amide bond, in which C═O stretch and N─H bending vibration modes can be detected at 1672 cm^−1^ wavenumber position. Thus, the contribution of 1672 cm^−1^ peak in SM can be of mixed origin, carbonyl stretching of phosphorylcholine and ceramide vibration modes. Phospholipids with different head groups such as phosphatidylethanolamine and phosphatidylserine have strong spectral features in the 1200 cm^−1^ wavenumber range. The 213 nm spectral library of individual biochemical standards can be found in the S1 dataset. The scope of this paper does not aim to fully assign vibrational/stretching modes of each biochemical component, which have been previously assigned elsewhere, for amino acids and nucleotides [67] and lipids [64], but to only briefly describe vibrational modes and positions of spectral markers that are relevant to the VSV virus. The present research mostly focuses on individual biochemical building blocks exhibiting strong resonance Raman effects. While a comprehensive analysis encompassing a wider range of individual biochemical species and complex structures would provide a more complete picture, this initial study aimed to establish the feasibility of utilizing Deep UV Resonance Raman spectroscopy in virus research. The current work serves as a foundation for future research that will incorporate a broader range of individual biochemical building blocks and higher‐order structures.

Next, to elucidate individual contributions and positions of biochemical standards in the virus spectrum, we rescaled every individual biomolecular spectrum. The magnitudes used for rescaling are presented in the table, middle panel of Figure 2, in which each individual biochemical component that can be found in the VSV virion is represented as a molecular weight fraction (ratio of the molecular weight of one component to the total molecular weight of all components in the VSV virion). The molecular weight fraction data for amino acids were calculated from the complete genome sequence of VSV (GenBank: J02428.1) and the molecular composition obtained by scanning transmission electron microscopy [68]. In brief, the VSV virion is composed of 5 proteins, G, M, N, P, and L, with different number of protein units per virion (G = 1205; M = 1258; N = 1826; p = 466; L = 50), the number of amino acid units obtained from each protein sequence was then multiplied with number of protein units per virion. Once total units for each amino acid were calculated, it was multiplied by their molecular weight and then divided by the total molecular weight of the VSV virion (265 MDa) to obtain their molecular weight fraction. The following procedure was applied to nucleotides and lipids as well. In the case of nucleotides, the process was much more straightforward; the molecular weight fraction of adenine, guanine, cytosine, and uracil was calculated from a reference GenBank sequence. To obtain the lipid composition of the VSV virion, the data were extracted from several sources [69, 70, 71, 72]. The complete calculated molecular weight fraction data used in this study can be found in the S2 dataset. The sum of our MW fraction data agrees with the total MW of the VSV virion and its biochemical components, which have been experimentally determined and previously published [68]. After completing molecular weight fraction calculation for each biocomponent, SNV‐normalized files of biomolecular standards are multiplied by their MW fraction part. The newly produced MW fraction‐biomolecular spectra are represented in the lowest panel of Figure 2. In this way, 17 biomolecular standards are positioned under the experimental VSV spectrum, indicating the position and degree of each biocomponent contributing to the VSV experimental spectrum. To further validate our approach, we averaged 17 MW fraction‐biomolecular spectra and defined them as the VSV stoichiometric spectrum. As can be seen in the lowest panel of Figure 2, the VSV stoichiometric spectrum is superimposable on the VSV experimental spectrum. Alternatively, and following spectra pretreatments and steps as mentioned above, a VSV stoichiometric spectrum can be generated using mol %, as described by Sapers et al. [73]. In our case, this involves multiplying the individual biocomponent by its mol %. For amino acids, we grouped resonant active aromatic amino acids F (4.56%), W (2.048%), and Y (4.51%), totaling 11.11%, and the remaining amino acids were assumed to have negligible DUV Raman cross‐sections, and their contribution was approximated by including a PBS buffer component (representing 88.89% of the total amino acid contribution). For lipids, we used the liposome spectrum, and for nucleotides: A (31.06%), G (21.78%), C (19.95%), U (27.2%). Finally, each biochemical group was multiplied by the corresponding total mass % of VSV virion (74.5% for amino acids, 21.1% for lipids, and 1.4% for nucleotides) [68].

With this approach, we managed to assign bands under the VSV spectrum more realistically and with real data than what is usually used, for instance, in peak fitting algorithms. While the relationship between spectral bands and biochemical compounds in the virion is inherently many‐to‐one due to vibrational overlaps, the 213 nm excitation leverages selective resonance enhancement to prioritize dominant spectral markers. We can see that the contribution of RNA is almost negligible because of the low percentage present in the total mass per virion (1.5%) and that the main contributions come from the amino acid and lipidic parts. Amino acid and lipid peaks overlap in the 1000 and 1200 cm^−1^ region, as well as in the 1550–1750 cm^−1^ range. However, the resonance effect provides two distinct, dominant spectral markers at positions 1610 and 1650 cm^−1^ that correspond to amino acid and lipidic components of the virion, respectively. As lipids can only be found in the viral envelope, we can suggest that the spectral marker at position 1650cm^−1^ is specifically related to that part.

### 2.3 Biochemical Characterization of Viruses

**FIGURE 3:**
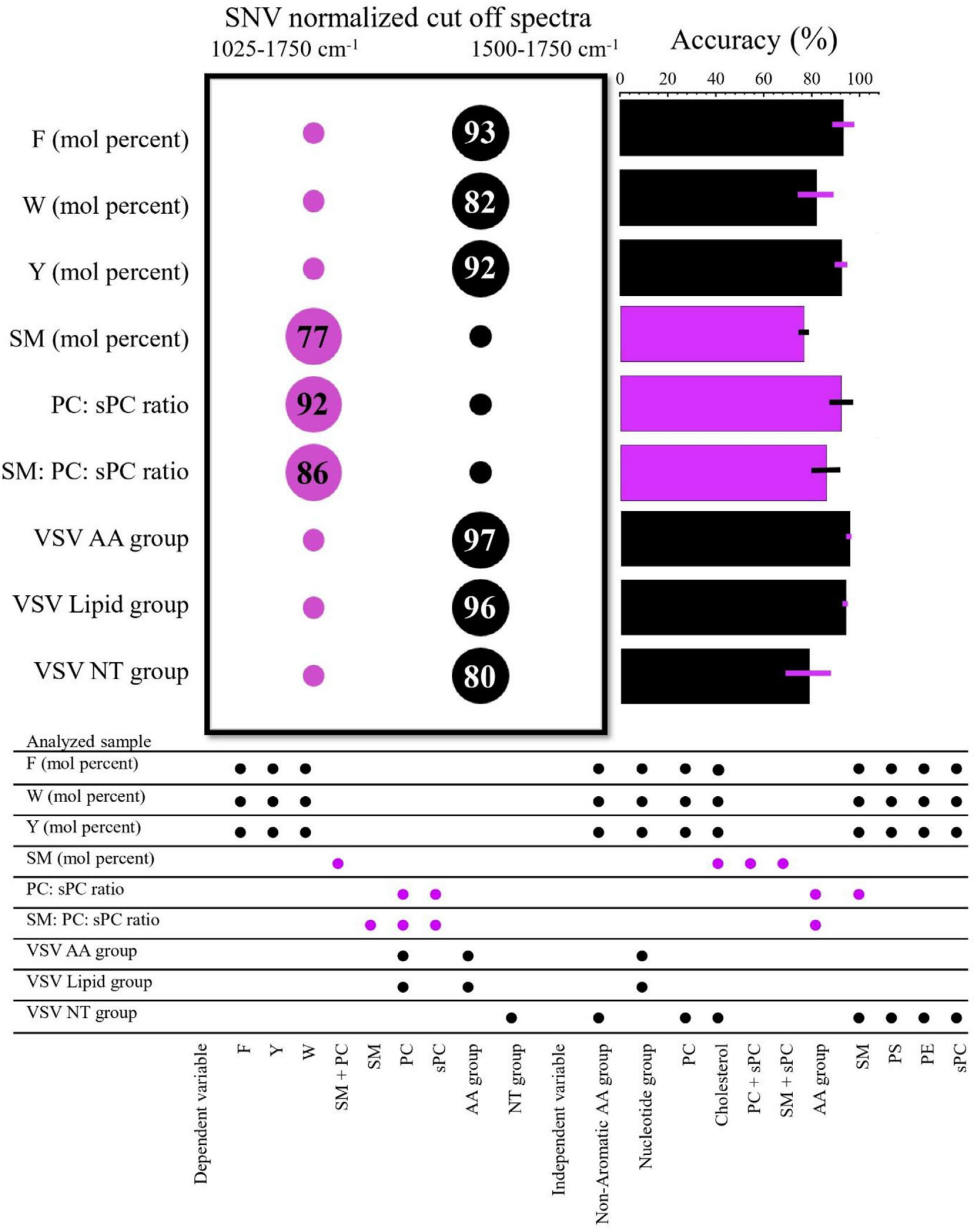
Performance and procedure of the biochemical characterization approach. The upper panel shows the performance of biochemical characterization in defined wavenumber ranges, magenta color for 1025–1750 cm^−1^, and black color for 1500–1750 cm^−1^ range. Values inside large circles indicate an average accuracy score tested on the VSV experimental spectrum, 3 stoichiometric models, and PBS buffer (*n* = 5 test models). Average accuracy scores are also represented as the height of the column, and error bars indicate the full range (minimum to maximum) of individual accuracy scores of tested models. A taller column indicates a higher average accuracy, and longer error bars suggest greater variability. The lower panel depicts the combination of dependent and independent variables necessary to accurately extract biochemical data by MLR analysis. Detailed statistical analyses that contain p‐values, the number of data points (N), Residual Sum of Squares (RSS), Adjusted R^2^, and ANOVA F‐statistics for all validation models across two spectral ranges (1025–1750 and 1500–1750 cm^−1^) can be found in S4 Dataset.

To assess to what extent DUVRR spectroscopy can be used as a tool for biochemical characterization of viruses in a label‐free approach, we have explored a method in which biochemical data can be directly extracted from the virus DUVRR spectrum. For this purpose, we have used a built‐in function of the Spectragryph software (developed by Dr. Friedrich Menges, Germany) [74], which operates on a multilinear regression algorithm (MLR) together with the singular value decomposition (SVD) method. This function calculates the amounts of known components in a mixture of unknown composition, in our case, the number of biomolecular components of the VSV virion. We first carried out a series of tests and found out that biochemical data extraction (biochemical characterization) performs well when the data is only baseline corrected and SNV normalized. Another important point is that the accuracy of biochemical data that can be extracted is improved when the function is applied to certain wavenumber ranges. The type of biochemical data that can be extracted in a specific wavenumber window is shown in Figure 3. The data have been encoded in such a way that the large circles represent biochemical data that can accurately be extracted, and vice versa, the data that cannot be extracted accurately appear as small‐sized circles. To consider an outcome positive, the extracted biochemical data (represented by large circles) must accurately reflect the known values used to create spectra for VSV stoichiometric (good agreement with VSV experimental), randomly created MW‐stoichiometric, molar fraction stoichiometric, and different from PBS buffer. The accuracy threshold for each model must be equal to or greater than 70%. The 70% accuracy threshold was established as an empirical selection criterion based on the consistent recovery rates observed across the five validation models. To ensure the mathematical robustness of this boundary, post‐hoc ANOVA and residual analysis were performed. These statistics reveal a clear distinction between viral models and the negative control (PBS buffer). While the high sensitivity of DUVRR results in significant correlations even within the PBS buffer (*p* < 0.05) due to water signals, the global model performance in the absence of a virion is characterized by a five‐fold increase in the Residual Sum of Squares (RSS) and a collapse of the F‐statistic. All five spectral models (VSV experimental, 3 pre‐defined stoichiometric models and their biochemical fraction data, and PBS) used to evaluate the biochemical characterization approach in this study can be found in the S3 dataset, with related statistics in the S4 dataset. In general, as a rule, when biochemical characterization is applied to a wider wavenumber range (1025–1750 cm^−1^) it is more suitable for lipid characterization, and when applied to a shorter range (1500–1750 cm^−1^) for amino acids. In cases where biochemical characterization performance is poorer, such as in the determination of SM and VSV NT group, the MW fraction of these components was found to be less than or equal to 1% in random MW stoichiometric and mol fraction model, respectively. Despite this model‐specific variation, the NT group generally represents ∼1.5% of the total virion mass. In these cases, p‐values may exceed 0.05 due to spectral crowding from dominant protein and lipid residues. To validate that the methodology remains sensitive to these minor constituents, additional testing was conducted using positive controls (100% NT, 25% Adenine, and VSV MW stoichiometric (Model 2) supplemented with 3% Adenine). These controls yielded high significance (*p* < 0.05), demonstrating that the extraction framework is capable of high‐sensitivity detection even when a component represents a minor fraction of the intact virion. In situations where the MW fraction or mol percent (number of units of one biocomponent within its biochemical group) of the biocomponent is greater than 2% or 5%, respectively, the accuracy is greatly improved and can reach up to 97% in the best cases. In this work, we developed a highly productive biochemical characterization approach that can be completed quickly, accurately, and resource‐wisely (effort and waste) to provide a fast response and biochemical data upon the emergence of a biological threat.

To facilitate the use, as well as encourage contributions and upgrades of our current approach, in the lower panel of Figure 3, we have provided the necessary information and combinations of files (S3 dataset) to perform biochemical characterization by MLR analysis.

**FIGURE 4:**
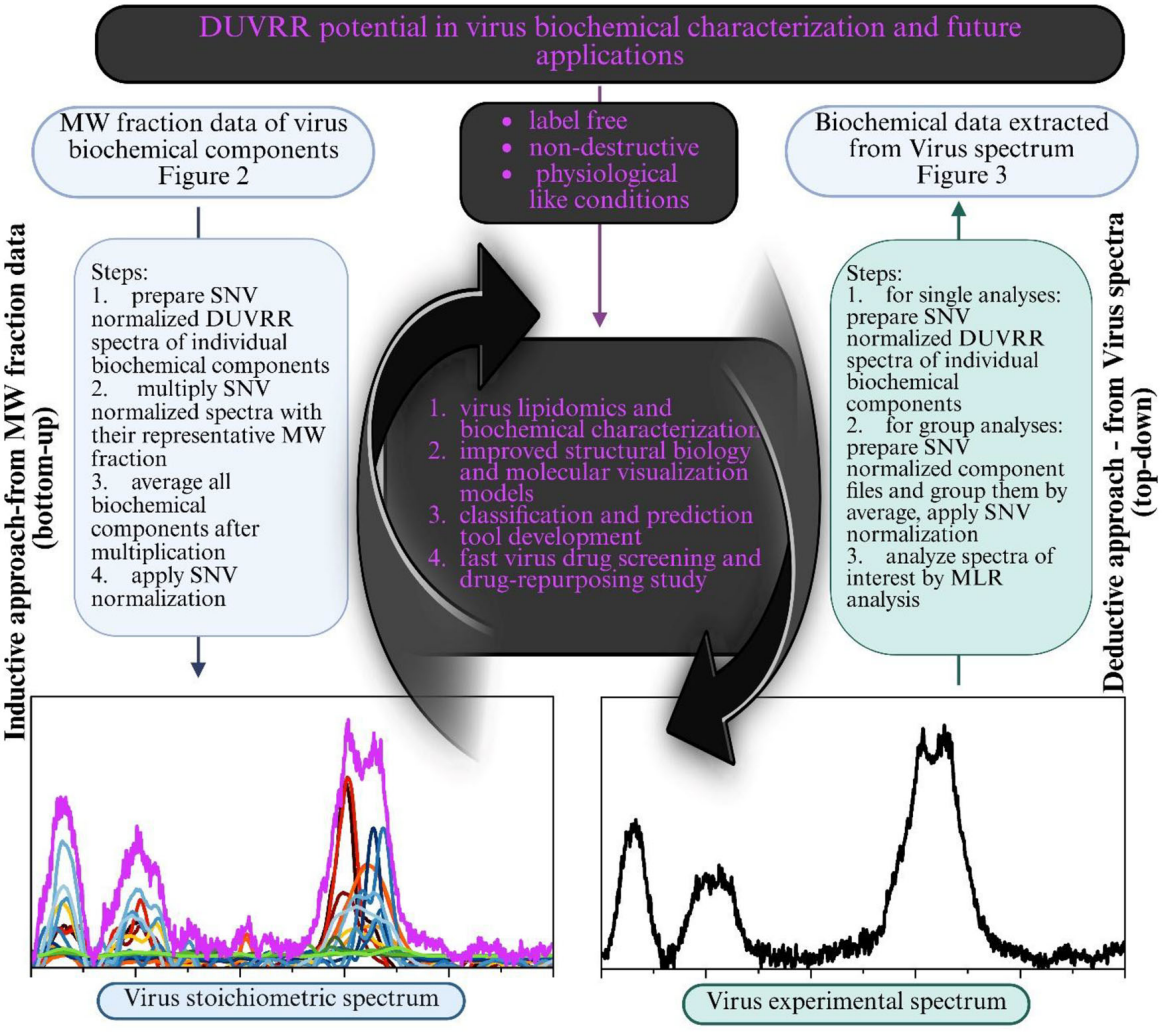
Graphical presentation of the Inductive and Deductive DUVRR approach and its further potential in virus research. On the left‐hand side are the necessary steps to perform a bottom‐up type of research, and on the right for top‐down. In the middle, we show possible future applications of DUVRR spectroscopy in virus research.

Next, Figure 4 provides an overview of biochemical characterization techniques applicable to DUVRR Virus Research, outlining the necessary data and steps for both bottom‐up and top‐down research approaches. According to the type of data that we can extract (Figure 3) and the way to create a virus spectrum from MW fraction data (Figure 2), we gave insight into DUVRR potential and possible future applications in the most probable virus research fields.

**FIGURE 5:**
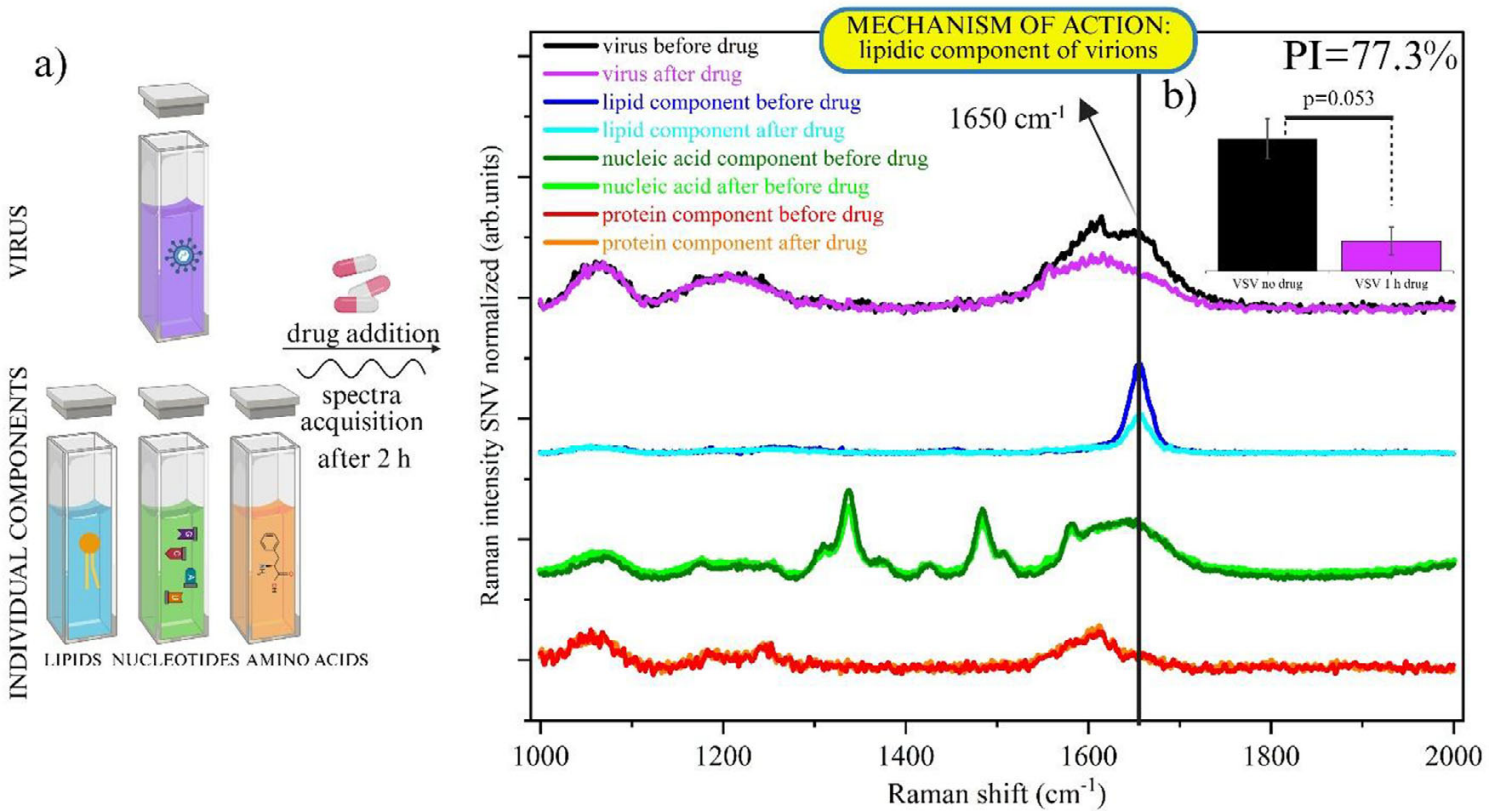
Assessment of antiviral susceptibility by DUVRR spectroscopy. (a) The Virus and individual biochemical standards were incubated with the antiviral drug HeE1‐17Y at a 1 µM concentration. After 2 h of drug incubation, the spectra of the virus and the individual components were collected. The VSV spectra before drug addition are presented in black and after drug addition in magenta color. Individual biocomponents before and after drug addition are represented for lipids in blue scale, nucleotides in green, and amino acids in red scale. (b) The virucidal effect of the drug was evaluated using plaque assay after incubating the drug with VSV virus for 1 h. The Percentage of the Inhibition (PI) was 77.3%, and statistical significance between groups was calculated via a two‐sample unpaired Student's t‐test (*n* = 2, with the exact p‐value reported above the bracket).

For instance, our individual contributions and expertise allow us to evaluate the premise of using DUVRR spectroscopy as a tool in the field of fast virus drug screening and drug repurposing studies. Therefore, to assess to what extent DUVRR can be used as a fast and reliable tool to determine the mechanism of antivirals, we exposed VSV to the Pyridobenzothiazolone derivative HeE1‐17Y with a described virucidal mechanism of action that targets the lipidic components of enveloped viruses, as previously characterized by our group through structural and biological assays [75]. The results are shown in Figure 5, in which the VSV virus sample and individual biomolecular standards have been incubated with the antiviral drug HeE1‐17Y for 2 h. We know that the peak at a position of 1650 cm^−1^ provides us information about the lipidic component of the virus envelope; therefore, we could hypothesize that the drug should cause spectral perturbations in that region. As can be seen, after 2 h of drug incubation, the peak at position 1650 cm^−1^ is fully decayed in the virus spectrum. By analyzing the effect of the drug on single components, we found only its activity against the lipid component, with main effect on 1650 cm^−1^ band. To assess the virucidal effect of the drug, we performed a plaque assay in which we incubated the drug with VSV. The results suggest that the drug abolished viral infectivity to 70%–90% after 1 h incubation.

In summary, this study presents a novel concept based on DUVRR measurements to characterize an enveloped mammalian RNA virus. We have shown how to create a stoichiometric virus spectrum by using the molecular weight fraction of individual biomolecular components of amino acids, lipids, and nucleotides. The comparison with the experimentally collected spectra of the VSV virus demonstrates the reliability and reproducibility of our method. We have built an accurate spectral library for the 213 nm wavelength of excitation useful for the structural elucidation of complex biological specimens such as high‐risk viruses. The procedure described in this work can be applied to other types of enveloped viruses with known protein and nucleotide sequences and known lipid composition (top‐down approach). When it is feasible to measure highly infective material (bottom‐up approach), we have explored how DUVRR can be used as a biochemical characterization strategy in which amino acid, lipid, and genomic parts of the virion can be determined with high accuracy in physiological‐like conditions and a label‐free approach. Finally, we gave a practical example of the application of DUVRR to the study of antiviral drugs. HeE1‐17Y and its mechanism of action on enveloped viruses were fully characterized by various methods to demonstrate targeting of the lipidic component of the virus [75]. Using DUVRR, we investigated the mechanism of action of HeE1‐17Y on lipids using a single instrument and operator within 2 h. This rapid, label‐free approach provides valuable information on the effects of antivirals on individual viral components under physiologically relevant conditions. In addition, databases of antivirals with known and unknown mechanisms of action can be created and classified into target groups (lipid, protein, nucleotide) by monitoring their effect on individual biomolecular components and whole viruses. By implementing the same procedure as mentioned above, Deep UV Resonance Raman can effectively be used as a first step, fast, and reliable drug discovery/repurposing strategic tool. However, while this DUVRR‐based approach provides a powerful tool for rapid biochemical characterization, several practical limitations remain. The current sensitivity of the technique requires high viral titers (approximately 1 mg/mL) to obtain high‐quality spectra with sufficient SNR ratio, as well as purified samples, which can impact result delivery time. Ideally, the fast response capability in a pandemic scenario would necessitate rapid upstream purification and concentration steps.

Finally, the simplicity of the sample preparation and analytical steps involved, together with the availability of miniaturized DUVRR spectrophotometers [76, 77] on the market, makes this technology well‐suited to be implemented in high‐containment biosafety level 3–4 laboratories as a tool of first choice to improve overall pandemic preparedness and countermeasure program.

## 3 Material and Methods

### 3.1 Virus Propagation and Purification

*The Vesicular Stomatitis Virus Indiana* (VSV) (ATCC‐1238), a biosafety level 2 microorganism, was propagated in the Vero E6 cell line (ATCC‐1586), which was cultured in Opti‐MEM medium (Thermo‐Fisher, UK) at 37°C with 5% CO_2_. Supernatants were harvested when full cytopathic effect was observed, prior to extensive cell detachment to minimize cellular debris contamination. Subsequent processing was performed as described by Moerdyk‐Schauwecker et al. [78]. In the first step, the supernatant was centrifuged at 3000 × g for 20 min to remove large cellular debris. Second, virus supernatant was transferred to ultracentrifuge tubes and was pelleted with a Beckman 60Ti type rotor at 71 000 × g and 4°C for 1 h. The viral pellet was resuspended in PBS buffer solution and centrifuged overnight in a 7%–60% discontinuous sucrose gradient at 130 000 × g and 4°C using a Beckman SW41 Ti rotor. Sucrose solutions were made in PBS buffer. Once the ultracentrifugation finished, the band containing the virus was removed from the centrifuge tube and diluted in PBS. The virus‐containing solution was pelleted twice at 130 000 × g and 4°C for 1 h using a Beckman 60Ti rotor to remove sucrose residues. The viral pellet was resuspended in PBS, and titers of infectious virus were determined by plaque assay on Vero E6 cells [79]. For DUVRR spectroscopy, the virus sample solutions were diluted to approximately 1 mg/mL in PBS buffer and quantified via UV absorbance at 280 nm.

### 3.2 Biochemical Standards

All biochemical standards were prepared in a final concentration of 10 mM in PBS solution. Amino acids: L‐tyrosine, L‐tryptophan, L‐phenylalanine, L‐alanine, glycine, L‐histidine, and L‐cysteine (Sigma, Germany) were directly prepared in PBS solutions. Nucleotides (Jena Bioscience, Germany) were diluted from a 100 mM stock solution in PBS. Lipids: L‐α‐phosphatidylcholine (PC), sphingomyelin (SM), L‐α‐phosphatidylserine (PS), cholesterol (CHOL), stearylamine (Sigma, Germany), and dipalmitoyl phosphocholine (DPPC), distearoyl phosphocholine (DSPC), dipalmitoyl phosphoethanolamine (DPPE, abbr. PE) (Avanti lipids, USA) were prepared as 100 mM stock solutions in methanol and diluted in PBS. In case of liposomes, they were prepared by the “Thin Film Hydration Method” reported by Bangham and Horne [80], in a ratio of PC‐65%, CHOLl‐30% and stearylamine‐5%. PC and stearylamine were diluted in methanol, and CHOL was diluted in a solution of methanol:chloroform with a 2:1 ratio. The lipid mixture was evaporated using a rotary evaporator and recovered with glass beads and PBS solution. After recovering, liposomes were briefly sonicated.

### 3.3 DUVRR Experimental Setup

DUVRR measurements were carried out at the BL10.2‐IUVS beamline of Elettra Sincrotrone Trieste (Italy), and the setup is described briefly by Rossi et al. [81]. The excitation wavelength of 213 nm was provided by a passively Q‐switched laser (FQSS 213‐Q, Crylas), and the power on the sample was about 0.3 mW. The Raman signal of the samples was collected in back‐scattered geometry, using an incident beam perpendicular to the sample, with the holder tilted at an angle of 0°–60° to minimize the influence of the elastic scattering line. The scattered light was analyzed via a single‐pass Czerny‐Turner spectrometer (Trivista 557, Princeton Instruments, 750 mm of focal length) equipped with a holographic grating at 3600 g/mm and detected using a UV‐enhanced CCD camera, thermo‐electrically cooled at −75°C. A 50 nm slit was selected for the optimum spectral resolution and signal intensity. The resolution was set at about 1.3 cm^−1^/pixel. The calibration of the spectrometer was performed using cyclohexane (spectroscopic grade, Sigma–Aldrich). The spectra were collected in vertical‐vertical (VV) polarization. All samples were measured in a Suprasil‐quartz cuvette under constant oscillation to prevent photodamage and ensure representative sampling. Each DUVRR spectrum was recorded in the range between 1000 and 2700 cm^−1^, with 20 s acquisition time and 20 accumulations. Each reported spectrum represents the average of three independent measurements, unless stated otherwise.

### 3.4 UVRR Spectral Treatment and Data Analysis

The collected Raman spectra were processed using Spectragryph software, then visualized and statistically analyzed with OriginLab software. First, the Raman data were truncated to the wavenumber region of interest 1000–2000 cm^−1^. Both spike removal and baseline correction were applied to the spectra. No spectral smoothing was applied to the spectra.

For the evaluation of the biochemical characterization procedure, SNV normalization was chosen. In SNV normalization, each spectrum is subtracted by its own mean and divided by the spectrum's standard deviation (each spectrum will have a mean of 0 and a standard deviation of 1). This type of normalization procedure corrects changes in optical path length, baseline, and light scattering and makes all spectra comparable in terms of intensities [82, 83]. For biochemical characterization analysis, we used analyze mixture function in Spectragryph software [74] that utilizes a multilinear regression algorithm (MLR) [84, 85] together with the singular value decomposition (SVD) method [86]. MLR is a statistical technique used to model the relationship between a dependent variable (predictions) and multiple independent variables (the constants), and SVD is a mathematical technique for decomposing a matrix, reducing dimensionality, and identifying and addressing collinearity.

### 3.5 Study of Mechanism of Action of HeE1‐17Y Antiviral Drug on Virus and Biochemical Standards

The antiviral pyridobenzothiazolone derivative HeE1‐17Y [75] was dissolved in DMSO and prepared as a 1 mM stock solution. To evaluate the effect of the antiviral drug on VSV virus and biochemical standards, the compound was preincubated at room temperature for 2 h at a final concentration of 1 µM. After 2 h, the samples were placed in a Suprasil‐quartz cuvette and measured in DUVRR conditions as mentioned above. The controls were left at RT and spiked with 0.1% DMSO (drug solvent). Infectivity (PFU/mL) of virus after 1 h of drug incubation was calculated by plaque assay on Vero E6 cells [79].

## Author Contributions

Conceptualization: D.R., C.M., A.M., and B.R.; Methodology: D.R., F.M., L.A., T.C., A.G., I.L., C.M., A.M., and B.R.; Investigation: D.R., F.M., and B.R.; Visualization: D.R., C.M., A.M., and B.R.; Supervision: I.L., C.M., A.M., and B.R.; Writing – original Draft: D.R., C.M., A.M., and B.R.; Writing – review & Editing: D.R., F.M., L.A., T.C., A.G., I.L., C.M., A.M., and B.R.

## Conflicts of Interest

The authors declare no conflicts of interest.

## Supporting information




**Supporting File**: smtd70845‐sup‐0001‐Data.zip.

## Data Availability

All data needed to evaluate the conclusions in the paper are present in the paper and/or the Supplementary Dataset.

## References

[1] M. Schoch‐Spana , A. Cicero , A. Adalja , et al., “Global Catastrophic Biological Risks: Toward a Working Definition,” Health Security 15 (2017): 323–328, 10.1089/hs.2017.0038. PMC5576209 28745924

[2] World Health Organization , Blueprint for R&D preparedness and response to public health emergencies due to highly infectious pathogens (World Health Organization, 2015).

[3] S. Drożdżal , J. Rosik , K. Lechowicz , et al., “An Update on Drugs with Therapeutic Potential for SARS‐CoV‐2 (COVID‐19) Treatment,” Drug Resistance Updates 59 (2021): 100794. 10.1016/j.drup.2021.100794 PMC8654464 34991982

[4] T. Dong , M. Wang , J. Liu , et al., “Diagnostics and Analysis of SARS‐CoV‐2: Current Status, Recent Advances, Challenges and Perspectives,” Chemical Science 14 (2023): 6149–6206, 10.1039/D2SC06665C. PMC10266450 37325147

[5] J. Baggen , M. Jacquemyn , L. Persoons , et al., “TMEM106B is a Receptor Mediating ACE2‐Independent SARS‐CoV‐2 Cell Entry,” Cell 186 (2023): 3427–3442.e22, 10.1016/j.cell.2023.06.005. PMC10409496 37421949

[6] A. D. Radford , D. Chapman , L. Dixon , J. Chantrey , A. C. Darby , and N. Hall , “Application of next‐Generation Sequencing Technologies in Virology,” Journal of General Virology 93 (2012): 1853–1868, 10.1099/vir.0.043182-0. PMC3709572 22647373

[7] T. P. Wörner , T. M. Shamorkina , J. Snijder , and A. J. R. Heck , “Mass Spectrometry‐Based Structural Virology,” Analytical Chemistry 93 (2021): 620–640. 10.1021/acs.analchem.0c04339 PMC7807421 33275424

[8] K. Zhou , Z. Si , P. Ge , J. Tsao , M. Luo , and Z. H. Zhou , “Atomic Model of Vesicular Stomatitis Virus and Mechanism of Assembly,” Nature Communications 13 (2022): 5980, 10.1038/s41467-022-33664-4. PMC9549855 36216930

[9] J. E. Voss , M.‐C. Vaney , S. Duquerroy , et al., “Glycoprotein Organization of Chikungunya Virus Particles Revealed by X‐Ray Crystallography,” Nature 468 (2010): 709–712, 10.1038/nature09555. 21124458

[10] L. Lecoq , M.‐L. Fogeron , B. H. Meier , M. Nassal , and A. Böckmann , “Solid‐State NMR for Studying the Structure and Dynamics of Viral Assemblies,” Viruses 12 (2020): 1069, 10.3390/v12101069. PMC7599928 32987909

[11] S. Payne , “Methods to Study Viruses,” Viruses: From Understanding to Investigation (Academic Press, 2017), pp. 37–52.

[12] E. Smith and G. Dent , Modern Raman Spectroscopy: A Practical Approach (John Wiley & Sons, 2019), 10.1002/9781119440598.

[13] D. A. Long , Raman spectroscopy, 1st ed (McGraw‐Hill Book Company, 1977), 310.

[14] J. Lukose , A. K. Barik , Mithun N , et al., “Raman Spectroscopy for Viral Diagnostics,” Biophysical Reviews 15 (2023): 199–221, 10.1007/s12551-023-01059-4. PMC10088700 37113565

[15] S. Jiang , Y. Xiao , Q. Li , L. Jiang , J. Wu , and Y. Li , “SERS Technology in Virus Detection: Advances, Challenges, and Future Perspectives,” Biosensors and Bioelectronics 290 (2025): 117902. 10.1016/j.bios.2025.117902 40916249

[16] J. Ye , Y.‐T. Yeh , Y. Xue , et al., “Accurate Virus Identification with Interpretable Raman Signatures by Machine Learning,” Proceedings of the National Academy of Sciences of the United States of America 119 (2022): 2118836119. 10.1073/pnas.2118836119 PMC9191668 35653572

[17] S. A. Asher , “UV Resonance Raman Spectroscopy for Analytical, Physical, and Biophysical Chemistry,” Analytical Chemistry 65 (1993): 59A–66A, 10.1021/ac00050a717. 8439006

[18] T. G. Spiro , Biological Applications of Raman Spectroscopy (Wiley, 1987).

[19] Z. Q. Wen and G. J. Thomas , “UV Resonance Raman Spectroscopy of DNA and Protein Constituents of Viruses: Assignments and Cross Sections for Excitations at 257, 244, 238, and 229 nm,” Biopolymers 45 (1998): 247–256, 10.1002/(SICI)1097-0282(199803)45:3<247::AID-BIP7>3.0.CO;2-R. 9465787

[20] S. Kaminaka , Y. Imamura , M. Shingu , T. Kitagawa , and T. Toyoda , “Studies of Bovine Enterovirus Structure by Ultraviolet Resonance Raman Spectroscopy,” Journal of Virological Methods 77 (1999): 117–123, 10.1016/S0166-0934(98)00153-0. 10092135

[21] Z. Q. Wen , S. A. Overman , and G. J. Thomas , “Structure and Interactions of the Single‐Stranded DNA Genome of Filamentous Virus Fd: Investigation by Ultraviolet Resonance Raman Spectroscopy,” Biochemistry 36 (1997): 7810–7820, 10.1021/bi970342q. 9201924

[22] Z. Q. Wen , A. Armstrong , and G. J. Thomas , “Demonstration by Ultraviolet Resonance Raman Spectroscopy of Differences in DNA Organization and Interactions in Filamentous Viruses Pf1 and Fd,” Biochemistry 38 (1999): 3148–3156, 10.1021/bi981965m. 10074370

[23] Z. Q. Wen and G. J. Thomas , “Ultraviolet‐Resonance Raman Spectroscopy of the Filamentous Virus Pf3: Interactions of Trp 38 Specific to the Assembled Virion Subunit,” Biochemistry 39 (2000): 146–152, 10.1021/bi992018w. 10625489

[24] Z. Q. Wen , S. A. Overman , P. Bondre , and G. J. Thomas , “Structure and Organization of Bacteriophage Pf3 Probed by Raman and Ultraviolet Resonance Raman Spectroscopy,” Biochemistry 40 (2001): 449–458, 10.1021/bi0018887. 11148039

[25] S. P. A. Fodor and T. G. Spiro , “Ultraviolet Resonance Raman Spectroscopy of DNA with 200‐266‐nm Laser Excitation,” Journal of the American Chemical Society 108 (1986): 3198–3205, 10.1021/ja00272a006.

[26] B. Rossi , M. Tortora , S. Catalini , et al., “Insight into the Thermal Stability of DNA in Hydrated Ionic Liquids from Multi‐Wavelength UV Resonance Raman Experiments,” Physical Chemistry Chemical Physics 23 (2021): 15980–15988, 10.1039/D1CP01970H. 34313275

[27] S. P. A. Fodor , R. P. Rava , T. R. Hays , and T. G. Spiro , “Ultraviolet Resonance Raman Spectroscopy of the Nucleotides with 266‐, 240‐, 218‐, and 200‐nm Pulsed Laser Excitation,” Journal of the American Chemical Society 107 (1985): 1520–1529, 10.1021/ja00292a012.

[28] R. S. Jakubek , J. Handen , S. E. White , S. A. Asher , and I. K. Lednev , “Ultraviolet Resonance Raman Spectroscopic Markers for Protein Structure and Dynamics,” TrAC Trends in Analytical Chemistry 103 (2018): 223–229, 10.1016/j.trac.2017.12.002. PMC7003616 32029956

[29] S. P. A. Fodor , R. A. Copeland , C. A. Grygon , and T. G. Spiro , “Deep‐Ultraviolet Raman Excitation Profiles and Vibronic Scattering Mechanisms of Phenylalanine, Tyrosine, and Tryptophan,” Journal of the American Chemical Society 111 (1989): 5509–5518, 10.1021/ja00197a001.

[30] S. A. Asher , M. Ludwig , and C. R. Johnson , “UV Resonance Raman Excitation Profiles of the Aromatic Amino Acids,” Journal of the American Chemical Society 108 (1986): 3186–3197, 10.1021/ja00272a005.

[31] J. M. Antosiewicz and D. Shugar , “UV–Vis Spectroscopy of Tyrosine Side‐Groups in Studies of Protein Structure. Part 2: Selected Applications,” Biophysical Reviews 8 (2016): 163–177, 10.1007/s12551-016-0197-7. PMC4884208 28510057

[32] H. Takeuchi , “UV Raman Markers for Structural Analysis of Aromatic Side Chains in Proteins,” Analytical Sciences 27, 1077–1086 (2011), 10.2116/analsci.27.1077. 22076333

[33] M. K. Eagleburger , “Deep Ultraviolet Resonance Raman Spectroscopy as a Tool for Biomolecule Analysis,” Doctoral diss, University of Missouri‐Columbia, (2017).

[34] C. Halsey , “Deep Ultraviolet Resonance Raman Spectroscopy Of Membrane Proteins,” (Doctoral diss., University of Missouri‐Columbia, 2012).

[35] Q. Wu , T. Hamilton , W. H. Nelson , S. Elliott , J. F. Sperry , and M. Wu , “UV Raman Spectral Intensities of E. coli and Other Bacteria Excited at 228.9, 244.0, and 248.2 nm,” Analytical Chemistry 73 (2001): 3432–3440, 10.1021/ac001268b. 11476245

[36] W. H. Nelson , R. Manoharan , and J. F. Sperry , “UV Resonance Raman Studies of Bacteria,” Applied Spectroscopy Reviews 27 (1992): 67–124, 10.1080/05704929208018270.

[37] R. M. Jarvis and R. Goodacre , “Ultra‐Violet Resonance Raman Spectroscopy for the Rapid Discrimination of Urinary Tract Infection Bacteria,” FEMS Microbiology Letters 232 (2004): 127–132, 10.1016/S0378-1097(04)00040-0. 15033230

[38] K. Gaus , P. Rösch , R. Petry , et al., “Classification of Lactic Acid Bacteria with UV‐Resonance Raman Spectroscopy,” Biopolymers 82 (2006): 286–290, 10.1002/bip.20448. 16421858

[39] S. Chadha , R. Manoharan , P. Moënne‐Loccoz , W. H. Nelson , W. L. Peticolas , and J. F. Sperry , “Comparison of the UV Resonance Raman Spectra of Bacteria, Bacterial Cell Walls, and Ribosomes Excited in the Deep UV,” Applied Spectroscopy 47 (1993): 38–43, 10.1366/0003702934048505.

[40] O. Zukovskaja , S. Kloß , M. G. Blango , et al., “UV‐Raman Spectroscopic Identification of Fungal Spores Important for Respiratory Diseases,” Analytical Chemistry 90 (2018): 8912–8918, 10.1021/acs.analchem.8b01038. 29956919

[41] N. M. Ralbovsky , V. Egorov , E. Moskovets , P. Dey , B. K. Dey , and I. K. Lednev , “Deep‐Ultraviolet Raman Spectroscopy for Cancer Diagnostics: A Feasibility Study with Cell Lines and Tissues,” Cancer Studies and Molecular Medicine – Open Journal 5 (2019): 1–10.

[42] D. Kurouski , R. P. Van Duyne , and I. K. Lednev , “Exploring the Structure and Formation Mechanism of Amyloid Fibrils by Raman Spectroscopy: A Review,” The Analyst 140 (2015): 4967–4980, 10.1039/C5AN00342C. 26042229

[43] I. K. Lednev , A. S. Karnoup , M. C. Sparrow , and S. A. Asher , “α‐Helix Peptide Folding and Unfolding Activation Barriers: a Nanosecond UV Resonance Raman Study,” Journal of the American Chemical Society 121 (1999): 8074–8086, 10.1021/ja991382f.

[44] K. M. Sanchez , T. J. Neary , and J. E. Kim , “Ultraviolet Resonance Raman Spectroscopy of Folded and Unfolded States of an Integral Membrane Protein,” The Journal of Physical Chemistry B 112 (2008): 9507–9511, 10.1021/jp800772j. 18588328

[45] F. D'Amico , P. Zucchiatti , K. Latella , et al., “Investigation of Genomic DNA Methylation by Ultraviolet Resonant Raman Spectroscopy,” Journal of Biophotonics 13 (2020): 202000150. 10.1002/jbio.202000150 32729213

[46] L. M. Almehmadi , S. V. Reverdatto , V. V. Ermolenkov , A. Shekhtman , and I. K. Lednev , “In Situ Stability Test for mRNA Vaccines Based on Deep‐UV Resonance Raman Spectroscopy,” Analytical Chemistry 96 (2024): 1003–1008. 10.1021/acs.analchem.3c01761 PMC10845005 38052070

[47] K. K. Sullivan , K. Wright , A. Kim , et al., “Deep‐UV Resonance Raman Spectroscopy Enables Direct Quantification of mRNA in Intact Lipid Nanoparticle Formulations,” Analytical Chemistry 98 (2026): 16704–16707, 10.1021/acs.analchem.6c02624. 42200382

[48] R. Esposito , D. Mirra , L. Sportiello , G. Spaziano , and B. D'Agostino , “Overview of Antiviral Drug Therapy for COVID‐19: Where Do We Stand?,” Biomedicines 10 (2022): 2815, 10.3390/biomedicines10112815. PMC9687182 36359334

[49] N. Drayman , J. K. DeMarco , K. A. Jones , et al., “Masitinib Is a Broad Coronavirus 3CL Inhibitor That Blocks Replication of SARS‐CoV‐2,” Science 373 (2021): 931–936, 10.1126/science.abg5827. PMC8809056 34285133

[50] Z. Jin , X. Du , Y. Xu , et al., “Structure of Mpro from SARS‐CoV‐2 and Discovery of Its Inhibitors,” Nature 582 (2020): 289–293, 10.1038/s41586-020-2223-y. 32272481

[51] D. E. Gordon , G. M. Jang , M. Bouhaddou , et al., “A SARS‐CoV‐2 Protein Interaction Map Reveals Targets for Drug Repurposing,” Nature 583 (2020): 459–468, 10.1038/s41586-020-2286-9. PMC7431030 32353859

[52] L. Riva , S. Yuan , X. Yin , et al., “Discovery of SARS‐CoV‐2 Antiviral Drugs through Large‐Scale Compound Repurposing,” Nature 586 (2020): 113–119, 10.1038/s41586-020-2577-1. PMC7603405 32707573

[53] E. C. López‐Díez , C. L. Winder , L. Ashton , F. Currie , and R. Goodacre , “Monitoring the Mode of Action of Antibiotics Using Raman Spectroscopy: Investigating Subinhibitory Effects of Amikacin on Pseudomonas aeruginosa,” Analytical Chemistry 77 (2005): 2901–2906. 10.1021/ac048147m 15859609

[54] A. Pistiki , O. Ryabchykov , A. Wagenhaus , et al., “Label‐Free Differentiation of Antimicrobial Resistance Groups Using Raman Spectroscopy,” Analytical Chemistry 98 (2026): 6523–6531, 10.1021/acs.analchem.5c03370. PMC12980480 41725368

[55] F. Moraca , S. Marzano , F. D'Amico , et al., “Ligand‐Based Drug Repurposing Strategy Identified SARS‐CoV‐2 RNA G‐Quadruplex Binders,” Chemical Communications 58 (2022): 11913–11916, 10.1039/D2CC03135C. 36196950

[56] V. W. Couling , P. Fischer , D. Klenerman , and W. Huber , “Ultraviolet Resonance Raman Study of Drug Binding in Dihydrofolate Reductase, Gyrase, and Catechol O‐Methyltransferase,” Biophysical Journal 75 (1998): 1097–1106, 10.1016/S0006-3495(98)77599-X. PMC1299784 9675211

[57] C. Domes , R. Domes , J. Popp , M. W. Pletz , and T. Frosch , “Ultrasensitive Detection of Antiseptic Antibiotics in Aqueous Media and Human Urine Using Deep UV Resonance Raman Spectroscopy,” Analytical Chemistry 89 (2017): 9997–10003. 10.1021/acs.analchem.7b02422 28840713

[58] C. Domes , J. Popp , S. Hagel , M. W. Pletz , and T. Frosch , “Towards Therapeutic Drug Monitoring of Antibiotic Levels—Analyzing the Pharmacokinetics of Levofloxacin Using DUV‐Resonance Raman Spectroscopy,” The Analyst 148 (2023): 3057–3064, 10.1039/D3AN00402C. 37272589

[59] R. Domes and T. Frosch , “Investigations on the Novel Antimalarial Ferroquine in Biomimetic Solutions Using Deep UV Resonance Raman Spectroscopy and Density Functional Theory,” Analytical Chemistry 95 (2023): 7630–7639, 10.1021/acs.analchem.3c00539. PMC10193365 37141178

[60] G. J. Letchworth , L. L. Rodriguez , and J. del C Barrera , “Vesicular Stomatitis,” The Veterinary Journal 157 (1999): 239–260, 10.1053/tvjl.1998.0303. 10328837

[61] G. Liu , W. Cao , A. Salawudeen , et al., “Vesicular Stomatitis Virus: From Agricultural Pathogen to Vaccine Vector,” Pathogens 10 (2021): 1092, 10.3390/pathogens10091092. PMC8470541 34578125

[62] F. D'Amico , F. Cammisuli , R. Addobbati , et al., “Oxidative Damage in DNA Bases Revealed by UV Resonant Raman Spectroscopy,” Analyst 140 (2015): 1477–1485. 10.1039/c4an02364a 25615720

[63] M. Harz , P. Rösch , and J. Popp , “Vibrational Spectroscopy—A Powerful Tool for the Rapid Identification of Microbial Cells at the Single‐Cell Level,” Cytometry Part A 75 (2009): 104–113, 10.1002/cyto.a.20682. 19156822

[64] K. Czamara , K. Majzner , M. Z. Pacia , K. Kochan , A. Kaczor , and M. Baranska , “Raman Spectroscopy of Lipids: A Review,” Journal of Raman Spectroscopy 46 (2015): 4–20, 10.1002/jrs.4607.

[65] H. G. M. Edwards , “Spectra‐Structure Correlations in Raman Spectroscopy,” Handbook of Vibrational Spectroscopy (John Wiley & Sons, 2002), pp. 1838–1871.

[66] C. Krafft , L. Neudert , T. Simat , and R. Salzer , “Near Infrared Raman Spectra of Human Brain Lipids,” Spectrochimica Acta Part A: Molecular and Biomolecular Spectroscopy 61 (2005): 1529–1535, 10.1016/j.saa.2004.11.017. 15820887

[67] J. Razzell Hollis , S. Sharma , W. Abbey , et al., “A Deep Ultraviolet Raman and Fluorescence Spectral Library of 51 Organic Compounds for the SHERLOC Instrument onboard Mars 2020,” Astrobiology 23 (2023): 1–23, 10.1089/ast.2022.0023. PMC9810352 36367974

[68] D. Thomas , W. W. Newcomb , J. C. Brown , et al., “Mass and Molecular Composition of Vesicular Stomatitis Virus: A Scanning Transmission Electron Microscopy Analysis,” Journal of Virology 54 (1985): 598–607, 10.1128/jvi.54.2.598-607.1985. PMC254833 2985822

[69] J. J. McSharry and R. R. Wagner , “Lipid Composition of Purified Vesicular Stomatitis Viruses,” Journal of Virology 7 (1971): 59–70, 10.1128/jvi.7.1.59-70.1971. PMC356078 5101092

[70] E. J. Patzer , N. F. Moore , Y. Barenholz , J. M. Shaw , and R. R. Wagner , “Lipid Organization of the Membrane of Vesicular Stomatitis Virus,” Journal of Biological Chemistry 253 (1978): 4544–4550, 10.1016/S0021-9258(17)30422-2. 207703

[71] L. Kalvodova , J. L. Sampaio , S. Cordo , C. S. Ejsing , A. Shevchenko , and K. Simons , “The Lipidomes of Vesicular Stomatitis Virus, semliki Forest Virus, and the Host Plasma Membrane Analyzed by Quantitative Shotgun Mass Spectrometry,” Journal of Virology 83 (2009): 7996–8003, 10.1128/JVI.00635-09. PMC2715782 19474104

[72] K. E. Havranek , J. M. Reyes Ballista , K. M. Hines , and M. A. Brindley , “Untargeted Lipidomics of Vesicular Stomatitis Virus‐Infected Cells and Viral Particles,” Viruses 14 (2021): 3, 10.3390/v14010003. PMC8778780 35062207

[73] H. M. Sapers , J. R. Hollis , R. Bhartia , et al., “The Cell and the Sum of Its Parts: Patterns of Complexity in Biosignatures as Revealed by Deep UV Raman Spectroscopy,” Frontiers in Microbiology 10 (2019): 679. 10.3389/fmicb.2019.00679 PMC6527968 31156562

[74] F. Menges , “Spectragryph—Optical Spectroscopy Software,” Version 1.2.17d, (2023), https://www.effemm2.de/spectragryph.

[75] R. Milan Bonotto , F. Bonì , M. Milani , et al., “Virucidal Activity of the Pyridobenzothiazolone Derivative HeE1‐17Y against Enveloped RNA Viruses,” Viruses 14 (2022): 1157, 10.3390/v14061157. PMC9228864 35746629

[76] W. J. Abbey , R. Bhartia , L. W. Beegle , et al., “Deep UV Raman Spectroscopy for Planetary Exploration: The Search for in Situ Organics,” Icarus 290 (2017): 201–214, 10.1016/j.icarus.2017.01.039.

[77] S. V. Bykov and S. A. Asher , “Solid State Vanadate Laser and 213 nm Rayleigh Rejection Filter Enable Miniaturized Deep Ultraviolet Raman Spectrometers,” Applied Spectroscopy 79 (2025): 345–348, 10.1177/00037028241280722. 39324202

[78] M. Moerdyk‐Schauwecker , S.‐I. Hwang , and V. Z. Grdzelishvili , “Analysis of Virion Associated Host Proteins in Vesicular Stomatitis Virus Using a Proteomics Approach,” Virology Journal 6 (2009): 166, 10.1186/1743-422X-6-166. PMC2770056 19821998

[79] R. Dulbecco and M. Vogt , “Plaque Formation and Isolation of Pure Lines with Poliomyelitis Viruses,” Journal of Experimental Medicine 99 (1954): 167–182, 10.1084/jem.99.2.167. PMC2180341 13130792

[80] A. D. Bangham and R. W. Horne , “Negative Staining of Phospholipids and Their Structural Modification by Surface‐Active Agents as Observed in the Electron Microscope,” Journal of Molecular Biology 8 (1964): 660–668, 10.1016/S0022-2836(64)80115-7. 14187392

[81] B. Rossi , C. Bottari , S. Catalini , F. D'Amico , A. Gessini , and C. Masciovecchio , “Synchrotron‐Based Ultraviolet Resonance Raman Scattering for Material Science,” Molecular and Laser Spectroscopy (Elsevier, 2020), pp. 447–482.

[82] R. J. Barnes , M. S. Dhanoa , and S. J. Lister , “Standard Normal Variate Transformation and De‐Trending of near‐Infrared Diffuse Reflectance Spectra,” Applied Spectroscopy 43 (1989): 772–777, 10.1366/0003702894202201.

[83] K. H. Liland , A. Kohler , and N. K. Afseth , “Model‐Based Pre‐Processing in Raman Spectroscopy of Biological Samples,” Journal of Raman Spectroscopy 47 (2016): 643–650, 10.1002/jrs.4886.

[84] G. James , D. Witten , T. Hastie , and R. Tibshirani , An Introduction to Statistical Learning (Springer, 2013).

[85] M. Kuhn and K. Johnson , Applied Predictive Modeling (Springer, 2013), 10.1007/978-1-4614-6849-3.

[86] G. H. Golub and C. F. Van Loan , Matrix Computations (JHU press, 2013).

